# Identification of Pathogenic *PKHD1* Variants in Infants with Autosomal Recessive Polycystic Kidney Disease from the Dhofar Region, Oman

**DOI:** 10.12688/f1000research.171123.2

**Published:** 2026-04-24

**Authors:** Intisar Al Alawi, Maha Al Awadi, Fatma Al Awaid, Joshua Pillai, Matthew Sampson, Juliana E. Arcila Galvis, Ashwaq Al Maimani, Zainab Al Hashmi, John A. Sayer

**Affiliations:** 1 National Genetic Centre, Ministry of Health, Muscat, Oman; 2 Sultan Qaboos Hospital, Ministry of Health, Salalah, Oman; 3 Division of Pediatric Nephrology, Boston Children's Hospital, Boston, MA, USA; 4 Harvard Medical School, Boston, Massachusetts, USA; 5 Biosciences Institute, Newcastle University, Newcastle Upon Tyne, UK; 6 Renal Services, Newcastle Upon Tyne Hospitals NHS Foundation Trust, Newcastle upon Tyne, England, UK; 7 NIHR Newcastle Biomedical Research Centre, Newcastle upon Tyne, England, UK

**Keywords:** PKHD1, Autosomal Recessive Polycystic Kidney Disease, Dhofar, Consanguinity, Genetic heterogeneity, Oman

## Abstract

**Background:**

Autosomal recessive polycystic kidney disease (ARPKD) is a rare, inherited disorder primarily affecting the kidneys and liver. Disease-causing variants in *PKHD1* lead to a disruption of the encoded protein fibrocystin/polyductin. This study aims to identify disease causing variants in *PKHD1* in families from the Dhofar region of Oman.

**Methods:**

We conducted a case series of six families with antenatal diagnoses of ARPKD and postnatal deaths. Genetic testing was performed on neonates using Sanger sequencing and next-generation sequencing (NGS) to detect variants in *PKHD1.* *In silico* analysis of mutational consequences was performed.

**Results:**

5 distinct homozygous variants in the *PKHD1* gene were identified, including three pathogenic frameshift variants (c.6111_6112delTT, c.7011dupT and c.9550dupT), a nonsense variant (c.340C>T) and a homozygous deletion spanning exons 58-60 of the *PKHD1.* These alleles have not been reported in previous studies. *In silico* modelling identified pathogenic alleles, predicted to lead to either truncated protein products or nonsense-mediated decay.

**Discussion:**

Our findings identify disease causing *PKHD1* variants in this genetically distinct Dhofar population, potentially due to factors such as geographical isolation, consanguinity, and founder effects. The identification of previously undescribed variants underscores the need for regional genetic studies in understanding ARPKD and its genotype-phenotype correlations.

**Conclusion:**

This study reveals distinct *PKHD1* disease-causing variants in the Dhofar region of Oman, contributing to the broader genetic understanding of ARPKD. These findings highlight the value of region-specific genetic research in identifying new disease causing variants.

## Introduction

Autosomal recessive polycystic kidney disease (ARPKD) is one of the most common recessively inherited cystic kidney disorders, with an estimated incidence ranging from 1 in 6,000 to 1 in 55,000 live births ( Zerres, Hansmann et al. 1988, Guay-Woodford, Bissler et al. 2014, Bergmann, Guay-Woodford et al. 2018). ARPKD most commonly presents in the neonatal and infantile period. It is characterized by progressive renal cystic dysplasia, which leads to enlarged, echogenic kidneys and varying degrees of renal impairment, as well as congenital hepatic fibrosis, which may progress to portal hypertension and its complications. Pulmonary hypoplasia due to oligohydramnios is a frequent and often life-threatening feature in perinatal presentations. While ARPKD is classically thought of as a disease of infancy and childhood, there is increasing recognition of later-onset and even adult presentations, in which renal and hepatic manifestations may be milder, slowly progressive, or discovered incidentally. The clinical spectrum therefore ranges from perinatal lethality to survival into adulthood with chronic kidney disease, portal hypertension, or combined hepatorenal involvement. While there is no definitive cure, current treatments focus on managing symptoms and delaying disease progression ( Guay-Woodford, Bissler et al. 2014).

Disease-causing variants in *PKHD1*, which encodes the fibrocystin/polyductin protein, impair the development of the kidney and bile ducts. Defects in fibrocystin lead to kidney cyst formation and malformation of bile ducts in the liver, resulting in polycystic kidneys and congenital hepatic fibrosis. Truncating mutations in *PKHD1* often result in severe neonatal outcomes, reflecting a genotype-phenotype correlation ( Burgmaier, Brinker et al. 2021).

**Figure 1.**
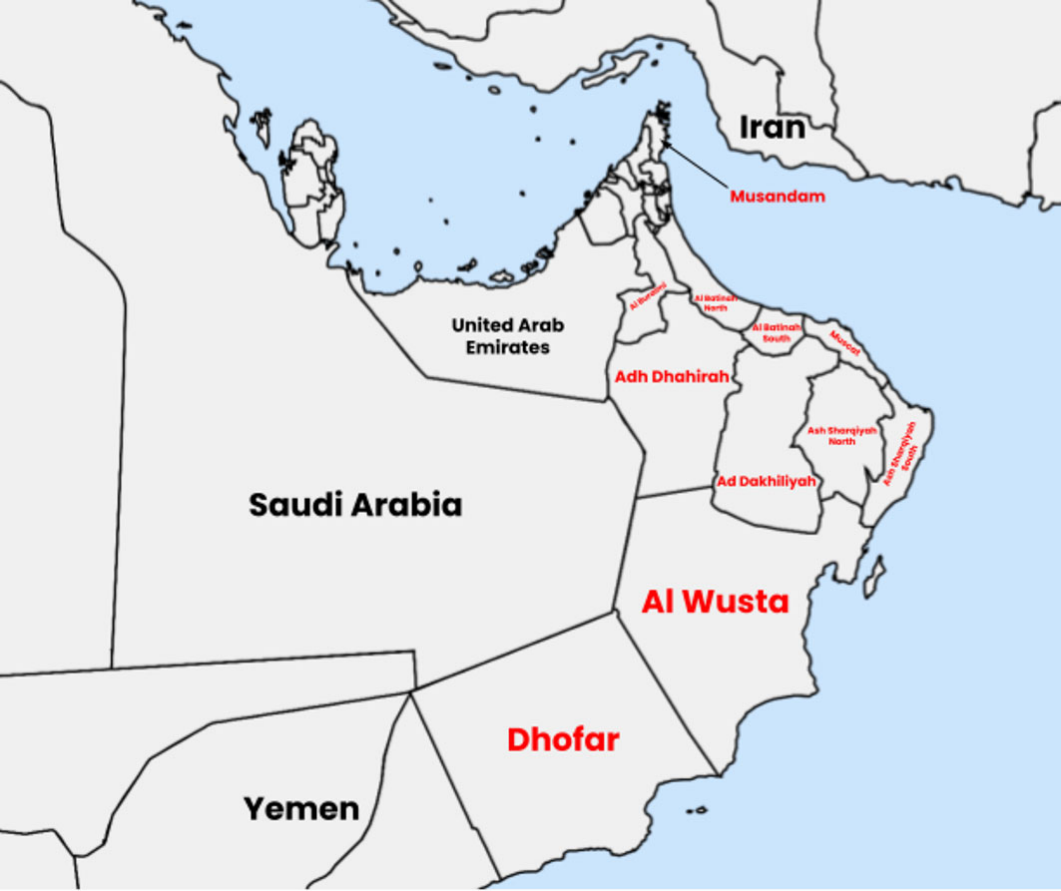
Geographical map of Oman and the Dhofar region. Map (drawn using https://www.mapchart.net/ and licensed under a Creative Commons Attribution-ShareAlike 4.0 International License.) showing Dhofar region (Dhofar Governorate) in the south of Oman. It is the largest of 11 governorates in Oman in terms of area with a population of around 500,000 people.

Although ARPKD has been widely studied in various populations, the genetic diversity of this disorder in Oman remains inadequately understood. Prior studies have identified several disease-causing variants in the *PKHD1* gene within the Omani population, but these studies did not focus on the Dhofar region ( Al Alawi, Molinari et al. 2020). The Dhofar region’s geographic isolation ( Figure 1), bordered by mountains and deserts, has fostered a unique demographic marked by consanguinity and minimal gene flow ( Rajab, Hamza et al. 2015).

Here we present a case series to investigate disease-causing *PKHD1* variants identified in neonates from six families in the Dhofar region. The study aims to expand the genetic landscape of ARPKD in Oman and contribute to the broader understanding of this rare genetic disorder.

## Methods

### Ethical approvals and patients’ inclusion and clinical evaluation

This case series includes six families from the Dhofar region of Oman who were referred to the National Genetic Centre at the Royal Hospital, Oman, following antenatal diagnoses of ARPKD between January 2023 and January 2025. All families had neonates with multiple congenital anomalies, including bilateral renal abnormalities, pulmonary hypoplasia, and oligohydramnios. This study was ethically approved by the Royal Hospital Scientific Research Ethical Committee, Ministry of Health, Oman (SRC/25647). For genetic studies of patients, written informed consent was provided by the parents of all affected neonates.

### Patient selection

The six families included in this study were referred to the National Genetic Centre at Royal Hospital, Oman, based on antenatal findings of congenital anomalies consistent with ARPKD. The clinical features included: oligohydramnios, bilateral kidney enlargement, pulmonary hypoplasia, and multiple congenital anomalies (e.g., dysmorphic features such as low-set ears, box-shaped head, and sandal gap deformity). The clinical presentation, including prenatal ultrasound findings, birth details, and postnatal progress, was recorded. Neonatal deaths occurred within 48 hours postpartum in all cases, which provided an opportunity for genetic analysis from post-mortem DNA samples. Additionally, clinical investigations included antenatal ultrasound scanning where the prenatal diagnosis of ARPKD was based on ultrasound findings showing enlarged and echogenic kidneys, absent bladder, and oligohydramnios. Postnatal clinical observations were performed on arrival at the Special Care Baby Unit (SCBU), where the neonates were monitored for respiratory distress (grunting, cyanosis, and decreased oxygen saturation), and vital signs were recorded. The clinical progress for each family such as gestational age at delivery, birth weight and vital signs (e.g., heart rate, blood pressure, oxygen saturation, and temperature) was documented.

### DNA isolation, library preparation and next generation sequencing

DNA samples for genetic analysis were extracted from cord blood/post-mortem blood specimens and archived at the National Genetic Centre for subsequent analysis. Clinical information was gathered from medical records, including antenatal ultrasound findings, birth outcomes, and postnatal management. Genetic testing was performed using both Sanger sequencing and next-generation sequencing (NGS) to identify mutations in the *PKHD1* gene ( Al Alawi, Al Salmi et al. 2019). For Sanger sequencing, target regions were amplified using AmpliTaq Gold 360 Master Mix kit (Applied Biosystems) using gene specific oligonucleotide primers. For NGS, a multiplex PCR approach amplified 4898 amplicons from 60 target genes (including *GANAB*, *HNF1B*, *NOTCH2*, *PKD1*, and *PKD2*, among others) using a custom QIAseq panel (Qiagen). Subsequent NGS was performed on an Illumina MiSeq system. The resulting data (vcf files) was processed through Variant Studio software to allow nucleotide variant and copy number variant calling. All variant nomenclature followed the standards set by the Human Genome Variation Society guidelines and confirmed via Sanger sequencing. *PKHD1* frameshift variants leading to predicted downstream nonsense alleles were modelled using MutationTaster2025 ( https://www.genecascade.org/MutationTaster2025/) and sequences are shown in Extended Data.

### Computational protein structure prediction

The wild-type structure of fibrocystin was predicted with AlphaFold3 ( Abramson, Adler et al. 2024) where the overall structure was of high confidence (pLDDT = 73.59). To model the frameshift and duplication variants, we predicted their complete structure and superimposed it on the wild-type. PyMOL was used for visualization ( Schrödinger and DeLano 2020).

## Results

### Clinical and molecular genetic findings

We analyzed six families with neonates affected by ARPKD. The clinical presentation of the affected neonates included signs of oligohydramnios, bilateral enlarged kidneys, pulmonary hypoplasia, and other dysmorphic features. All neonates died within 48 hours postpartum, with no survival beyond seven days. Table 1 illustrates the clinical presentation and outcomes of the affected neonates. The clinical progress for each family such as gestational age at delivery, birth weight and vital signs (e.g., heart rate, blood pressure, oxygen saturation, and temperature) was documented. The gestational age at delivery in our families ranged between 32 and 36 weeks, while the birth weight ranged from 2.3 kg to 3.4 kg. Table 2 summarizes these critical postnatal data.

**Table 1.** Clinical presentation and outcomes of affected neonates.

Family	Gestational age (weeks)	Birth weight (kg)	Key clinical features	*PKHD1* variant	Outcome
Family 1, affected female	34	2.36	Oligohydramnios, bilateral renal enlargement, pulmonary hypoplasia, weak cry, gasping, cyanosis, dysmorphic features.	Homozygous c.7011dupT; p.(Gly2338Trpfs*41) Chr6:51887230_51887231insA (GRCh38)	Expired within 48 hours
Family 2, affected female	33	2.36	Multiple congenital anomalies, oligohydramnios, breech presentation, pulmonary hypoplasia, bilateral kidney masses.	Homozygous c.7011dupT; p.(Gly2338Trpfs*41) Chr6:51887230_51887231insA (GRCh38)	Expired within 48 hours
Family 3, affected male	35	2.40	Polycystic kidneys, pulmonary hypoplasia, pneumothorax, dysmorphic features.	Homozygous c.6111_6112delTT; p.(Leu2039fs*13) Chr6:51934120_51934121delAA (GRCh38)	Expired within 7 hours
Family 4	32	2.30	Multiple congenital anomalies, bilateral palpable kidney masses, dysmorphic features.	Homozygous deletion exons 58-60 of *PKHD1*	Expired within 24 hours
Family 5	36	2.36	Oligohydramnios, enlarged kidneys, pulmonary hypoplasia, dysmorphic features.	Homozygous c.9550dupT; p.(Tyr3184Leufs*18) Chr6:51748065_51748066insA (GRCh38)	Expired within 22 hours
Family 6	32	3.40	Oligohydramnios, enlarged kidneys, pulmonary hypoplasia, dysmorphic features.	Homozygous c.340C>T; p.Q114* Chr6:52079950G>A (GRCh38)	Expired within 48 hours

*PKHD1* NCBI Reference sequence NM_138694.4

**Table 2.** Postnatal vital signs for affected neonates.

Family	Heart rate (bpm)	Blood pressure (mmHg)	Oxygen saturation (%)	Respiratory rate (bpm)	Temperature (°C)
Family 1	140	73/42	70	70	36.2
Family 2	149	73/42	75	60	36.0
Family 3	147	73/42	70	70	36.0
Family 4	130	68/40	65	60	36.5
Family 5	140	72/40	65	65	36.2
Family 6	N/A	N/A	N/A	N/A	N/A

**Figure 2.**
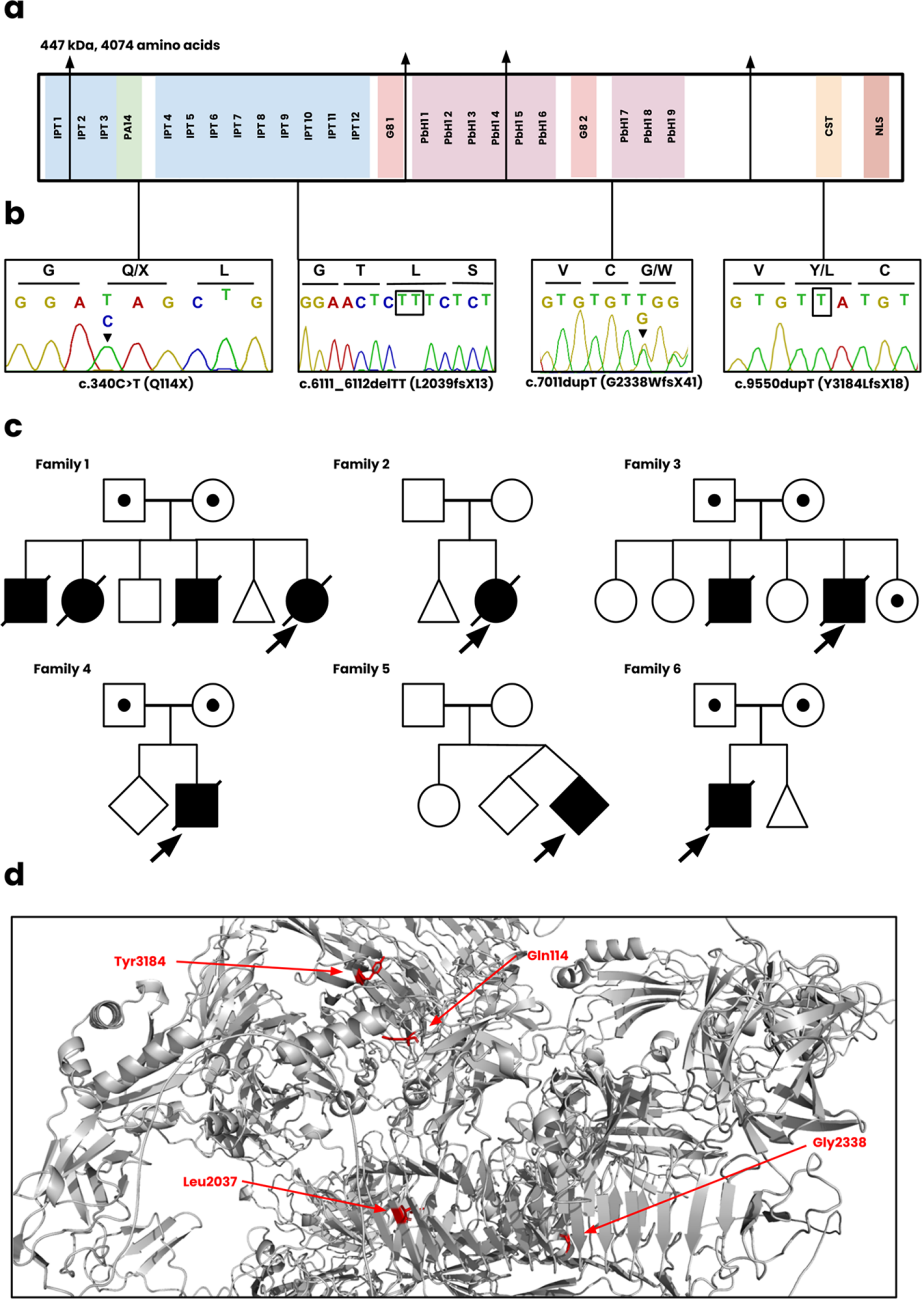
Genetic analysis of novel *PKHD1* variants. **(a)** The functional regions of fibrocystin, including the IPT, PA14, G8, PbH1, CST, and NLS domains. **(b)** Sanger sequencing of the c.340C>T, c.6111_6112delTT, c.7011dupT, c.9550dupT variants. The sequence for c.7011dupT of the patient is unavailable and instead displayed for the heterozygote parent. **(c)** The pedigree diagrams for all six Omani families. Squares, males; Circles, females; Shaded, affected. **(d)** Position of novel mutations on the wild-type structure of fibrocystin.

Each of the six families described had a molecular genetic diagnosis of ARPKD secondary to biallelic *PKHD1* disease causing variants ( Figure 2) and are summarized below.

Family 1 included a female infant born at 34 weeks of gestation to consanguineous parents presented antenatally with bilateral enlarged kidneys and pulmonary hypoplasia. The infant died within 48 hours. Genetic testing identified a homozygous frameshift mutation, *PKHD1* c.7011dupT; p.(G2338WfsX41), classified as likely pathogenic. There was a previous history of 3 neonatal deaths with similar clinical presentations.

Family 2 included a female infant, born at 33 weeks of gestation via breech delivery, had bilateral enlarged kidneys and pulmonary hypoplasia and died within 48 hours. Genetic analysis confirmed a homozygous frameshift mutation, *PKHD1* c.7011dupT; p.(G2338WfsX41). There was a previous history of an early miscarriage.

Family 3 included a male infant, born at 35 weeks of gestation, was diagnosed antenatally with polycystic kidneys and suspected Potter syndrome. The neonate died within 7 hours due to spontaneous pneumothorax. Genetic analysis confirmed a frameshift mutation (not present in gnomAD, ClinVar, Leiden Open Variation Database) *PKHD1*: c.6111_6112delTT; p.(L2039fsX13).

Family 4 included a male infant born at 32 weeks of gestation to consanguineous parents presented with bilateral enlarged kidneys and other dysmorphic features. Genetic analysis revealed a novel homozygous deletion spanning exons 58-60 of the *PKHD1* gene, classified as likely pathogenic.

Family 5 included a second twin from a consanguineous couple, born at 36 weeks, presented with oligohydramnios and bilateral kidney enlargement. The neonate died within 22 hours postpartum. A novel frameshift variant, *PKHD1* c.9550dupT; p.(Y3184LfsX18), was identified.

Family 6: A male infant was born at 32 weeks of gestation to consanguineous parents presented with oligohydramnios and bilateral kidney enlargement. A homozygous nonsense variant, *PKHD1* c.340C>T; p.(Q114*) was identified.

### *In silico* modelling of novel *PKHD1* variants

**Figure 3.**
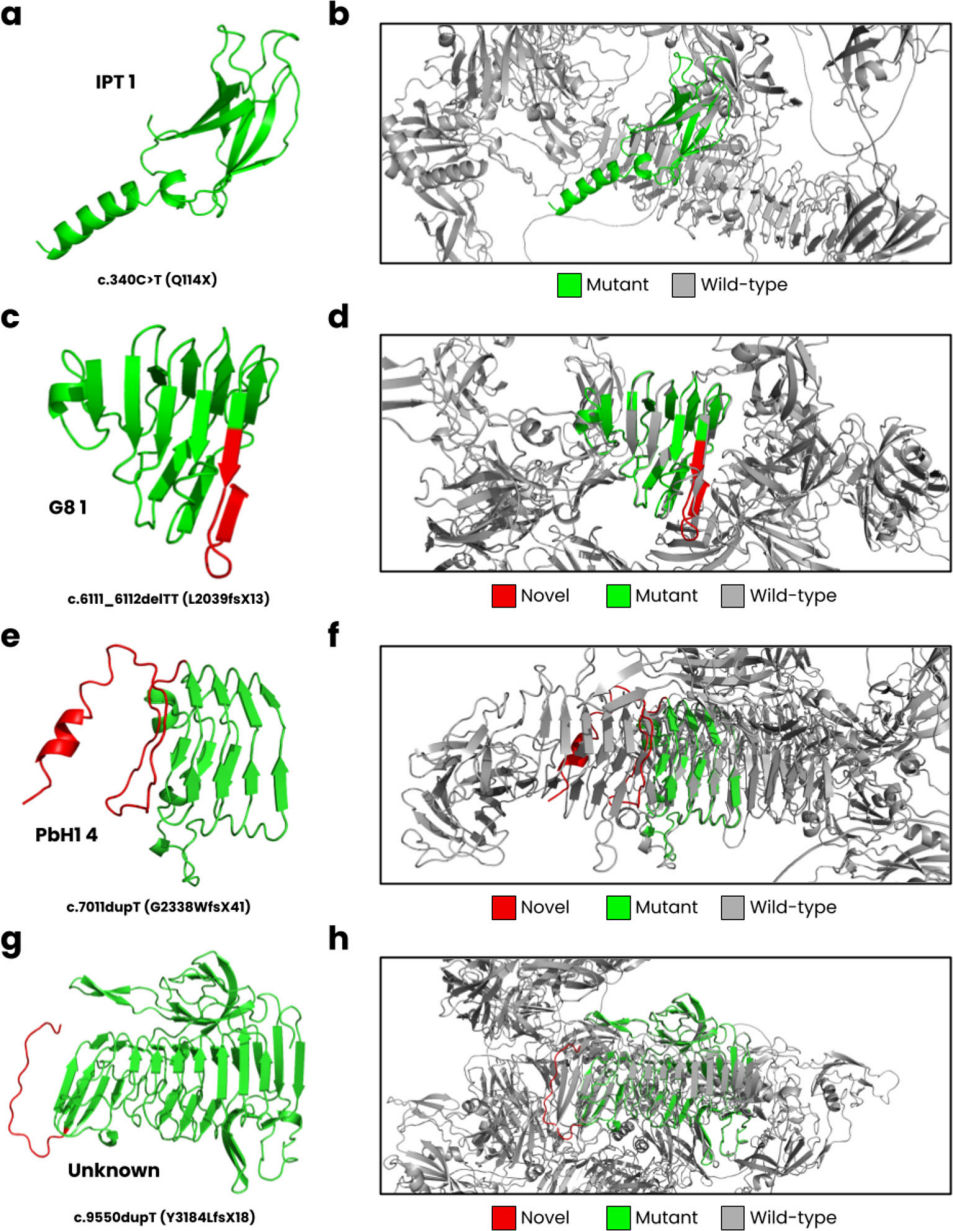
*In silico* modelling of *PKHD1* variants. **(a)** The c.340C>T mutation is predicted to cause a Q114X nonsense variant, **(b)** where the mutant structure only partially encodes for the IPT one domain compared to the wild-type structure. **(c)** The c.6111_6112delTT leads to L2039fsX13, impacting the G8 1 domain, and **(d)** encoding nearly half of the wild-type structure. **(e)** The c.7011dupT cause a G2338WfsX41 frameshift, **(f )** where PbH1 is partially lost. **(g)** Lastly, the c.9550dupT leads to Y3184LfsX18, encoding an unknown domain of *PKHD1* **(h)** close to the C-terminal region.

In Figure 3, we have displayed the predicted protein structures for the five novel *PKHD1* variants superimposed onto the wild-type fibrocystin structure. The c.340C>T results in a nonsense Q114X variant that encodes for the complete IPT 1 domain ( Figure 3a) but causes a loss of most of its wild-type structure ( Figure 3b). The c.6111_6112delTT is predicted to cause a L2039fsX13 frameshift, impacting the G8 1 domain ( Figure 3c). The wild-type G8 1 domain is composed of 10 β-strands and one α-helix ( He, Liu et al. 2006). In our structural model ( Figure 3d), we observed these structural characteristics, but an additional β-strand is formed resulting from the frameshift. The c.7011dupT mutation is predicted to result in a G2338WfsX41 frameshift, where the PbH1 domain is affected ( Figure 3e). The wild-type PbH1 domain is composed of 3 β-strands stacked into six repeats ( Mayans, Scott et al. 1997). In the mutant structure, PbH1 1 to PbH1 4 is normal, but the frameshift results in a loss of the remaining two repeats and instead a structure composed of a loop and α-helix is formed ( Figure 3f). Lastly, the c.9550dupT (Y3184LfsX18) is located on an unknown region of fibrocystin ( Figure 3g), where the frameshift does not form secondary structure, and the C-terminal region is lost ( Figure 3h).

## Discussion

The universal presence of pulmonary hypoplasia in these neonates aligns with ARPKD’s hallmark features and is often associated with Potter syndrome due to oligohydramnios ( Guay-Woodford, Bissler et al. 2014). The severity of oligohydramnios and lung underdevelopment could explain the early mortality observed in these cases. Despite genetic heterogeneity, the clinical presentations were remarkably consistent, with common features such as bilateral kidney enlargement and dysmorphic features. This could point to a shared pathophysiological mechanism linked to the *PKHD1* mutations in this population.

This case series highlights the identification of five distinct mutations in the *PKHD1* gene in neonates from the Dhofar region of Oman. The presence of a recurrent variant (c.7011dupT), a nonsense variant and three other novel variants (c.6111_6112delTT, c.9550dupT, and a large deletion) suggests significant genetic heterogeneity within the region. Notably, these alleles were not previously reported in studies of ARPKD in other parts of Oman ( Al Alawi, Molinari et al. 2020), indicating that the Dhofar population may harbor unique genetic variants. While previous studies of ARPKD in Oman have focused on more populous regions, this study uncovers significant genetic diversity within the Dhofar population. The identification of novel mutations, including c.6111_6112delTT and c.9550dupT, points to the complex genetic architecture of ARPKD in Oman, which may be influenced by regional variations in genetic isolation and migration patterns.

The Dhofar region, the southernmost governate of Oman, is geographically isolated by mountains and deserts, which likely restricts gene flow from neighboring populations. This isolation can contribute to the accumulation of distinct genetic mutations, as observed in the c.7011dupT allele in two families, who are likely to be related. This was also true for a *CFTR* allele reported in patients with cystic fibrosis that was also specific to this distinct geographical region ( Al Oraimi, Al Shidhani et al. 2022). The high rate of consanguinity in Dhofar (up to 68%, ( Al Oraimi, Al Shidhani et al. 2022)) likely enhances the likelihood of genetic drift and the founder effect, potentially explaining the presence of this mutation in multiple families.

## Conclusion

This study identifies five distinct *PKHD1* mutations in neonates from the Dhofar region of Oman, contributing to the genetic understanding of ARPKD. Our findings underscore the need for regional studies to map Oman’s genetic diversity. Further research, including larger studies involving other regions of Oman, is necessary to better understand the molecular basis of ARPKD in this population and to inform genetic counseling and management strategies for affected families.

## Data Availability

No underlying data associated with this article. Figshare: Extended data 1. https://doi.org/10.6084/m9.figshare.30188437.v1. This project contains the following extended data:
• Supplemental Material_PKHD1.docx Data are available under the terms of the Creative Commons Attribution 4.0 International license (CC-BY 4.0). Details of sequence variants and clinical information have been deposited at Leiden Open Variation Database 3.0 ( https://www.lovd.nl) ( Fokkema, Kroon, et al. 2021).
•
https://databases.lovd.nl/shared/individuals/00466658
•
https://databases.lovd.nl/shared/individuals/00466578
•
https://databases.lovd.nl/shared/individuals/00466580
•
https://databases.lovd.nl/shared/individuals/00466582
•
https://databases.lovd.nl/shared/individuals/00466585
•
https://databases.lovd.nl/shared/individuals/00466581

## References

[ref1] Abramson J Adler J Dunger J : Accurate structure prediction of biomolecular interactions with AlphaFold 3. *Nature.* 2024;630(8016):493–500. 10.1038/s41586-024-07487-w 38718835 PMC11168924

[ref2] Al Alawi I Al Salmi I Al Rahbi F : Molecular Genetic Diagnosis of Omani Patients With Inherited Cystic Kidney Disease. *Kidney Int Rep.* 2019;4(12):1751–1759. 10.1016/j.ekir.2019.08.012 31844813 PMC6895654

[ref3] Al Alawi I Molinari E Al Salmi I : Clinical and genetic characteristics of autosomal recessive polycystic kidney disease in Oman. *BMC Nephrol.* 2020;21(1):347. 10.1186/s12882-020-02013-2 32799815 PMC7429752

[ref4] Al Oraimi S Al Shidhani K Al Harthi H : Prevalence and Characteristics of Cystic Fibrosis in Omani Children: A Multi-center Cross-sectional Study. *Oman Med. J.* 2022;37(6):e444. 10.5001/omj.2022.101 36458240 PMC9627948

[ref5] Bergmann C Guay-Woodford LM Harris PC : Polycystic kidney disease. *Nat. Rev. Dis. Primers.* 2018;4(1):50. 10.1038/s41572-018-0047-y 30523303 PMC6592047

[ref6] Burgmaier K Brinker L Erger F : Refining genotype-phenotype correlations in 304 patients with autosomal recessive polycystic kidney disease and PKHD1 gene variants. *Kidney Int.* 2021;100(3):650–659. 10.1016/j.kint.2021.04.019 33940108

[ref7] Fokkema IF Kroon M López Hernández JA : The LOVD3 platform: efficient genome-wide sharing of genetic variants. *Eur. J. Hum. Genet.* 2021;29:1796–1803. 10.1038/s41431-021-00959-x 34521998 PMC8632977

[ref8] Guay-Woodford LM Bissler JJ Braun MC : Consensus expert recommendations for the diagnosis and management of autosomal recessive polycystic kidney disease: report of an international conference. *J. Pediatr.* 2014;165(3):611–617. 10.1016/j.jpeds.2014.06.015 25015577 PMC4723266

[ref9] He QY Liu XH Li Q : G8: a novel domain associated with polycystic kidney disease and non-syndromic hearing loss. *Bioinformatics.* 2006;22(18):2189–2191. 10.1093/bioinformatics/btl123 16632497

[ref10] Mayans O Scott M Connerton I : Two crystal structures of pectin lyase A from Aspergillus reveal a pH driven conformational change and striking divergence in the substrate-binding clefts of pectin and pectate lyases. *Structure.* 1997;5(5):677–689. 10.1016/S0969-2126(97)00222-0 9195887

[ref11] Rajab A Hamza N Al Harasi S : Repository of mutations from Oman: The entry point to a national mutation database. *F1000Res.* 2015;4:891. 10.12688/f1000research.6938.1 26594346 PMC4648203

[ref12] Schrödinger L DeLano W : PYMOL. 2020. Reference Source

[ref13] Zerres K Hansmann M Mallmann R : Autosomal recessive polycystic kidney disease. Problems of prenatal diagnosis. *Prenat. Diagn.* 1988;8(3):215–229. 10.1002/pd.1970080308 3287366

