# Supplementary material for: Identification of Pathogenic PKHD1 Variants in Infants with Autosomal Recessive Polycystic Kidney Disease from the Dhofar Region, Oman
Source: F1000Res. 2026 Apr 24;14:1212. Originally published 2025 Nov 5. [Version 2] doi: 10.12688/f1000research.171123.2 (PMC12856244; doi:10.12688/f1000research.171123.2)
Supplement: Peer review history

# Reviewer report: Reviewer response for version 1

**DOI:** 10.5256/f1000research.188677.r450003

**Published:** 2026-01-29

**Authors:** Consolato M Sergi

**Affiliations:** 1 Laboratory Medicine and Pathology, University of Alberta, Edmonton, Alberta, Canada; 2 Children’s Hospital of Eastern Ontario, Ontario, Canada

Infants with autosomal recessive polycystic kidney disease (ARPKD) in the Dhofar region of Oman display unique, previously unreported homozygous PKHD1 mutations (e.g., c.6111_6112delTT, c.7011dupT, c.9550dupT, c.340C>T, and deletion of exons 58-60). These variations, frequently associated with consanguinity and founder effects, generally result in shortened proteins or severe, early-onset diseases. Principal Discoveries Regarding PKHD1 in ARPKD Patients from Dhofar/Oman.

 The elevated prevalence of ARPKD in this location is ascribed to significant levels of consanguinity, geographical seclusion, and founder effects. These variations result in severe, early-onset ARPKD, frequently associated with a dismal prognosis, encompassing prenatal diagnosis and postnatal mortality. Given the detection of distinct, consistent variants throughout the population, targeted PCR investigation of these specific alleles is recommended for diagnosis. Al-Alawi et al. describe five unique homozygous variations in the *PKHD1* gene. These variants were discovered, comprising three pathogenic frameshift variants (c.6111_6112delTT, c.7011dupT, and c.9550dupT), a nonsense variant (c.340C>T), and a homozygous deletion encompassing exons 58-60 of the PKHD1. These alleles have not been documented in prior research. In silico modeling showed either shortened protein products or nonsense-mediated degradation. Their research confirms the geographical isolation, consanguinity, and founder effects. These findings support the significance of region-specific genomic studies in uncovering novel pathogenic mutations.

Is the work clearly and accurately presented and does it cite the current literature?

Yes

If applicable, is the statistical analysis and its interpretation appropriate?

Yes

Are all the source data underlying the results available to ensure full reproducibility?

Yes

Is the study design appropriate and is the work technically sound?

Yes

Are the conclusions drawn adequately supported by the results?

Yes

Are sufficient details of methods and analysis provided to allow replication by others?

Yes

Reviewer Expertise:

My group and I work on hepatorenal fibrocystic diseases.

I confirm that I have read this submission and believe that I have an appropriate level of expertise to confirm that it is of an acceptable scientific standard.

## References

[1] : Immunohistochemical detection of polyductin and co‐localization with liver progenitor cell markers during normal and abnormal development of the intrahepatic biliary system and in adult hepatobiliary carcinomas. *Journal of Cellular and Molecular Medicine* .2009;13(7) : 10.1111/j.1582-4934.2008.00519.x 1279-1290 10.1111/j.1582-4934.2008.00519.x PMC449614219292732

[2] : Successful transplantation in a child with rapid progression of autosomal recessive polycystic kidney disease associated with a novel mutation. *Pediatric Transplantation* .2006;10(3) : 10.1111/j.1399-3046.2005.00464.x 362-366 10.1111/j.1399-3046.2005.00464.x 16677362

[3] : Molecular pathology and genetics of congenital hepatorenal fibrocystic syndromes. *Journal of Medical Genetics* .2003;40(5) : 10.1136/jmg.40.5.311 311-319 10.1136/jmg.40.5.311 PMC173546012746391

# Reviewer report: Reviewer response for version 1

**DOI:** 10.5256/f1000research.188677.r447908

**Published:** 2026-01-13

**Authors:** Hana Yang

**Affiliations:** 1 Mayo Clinic Minnesota, Rochester, Minnesota, USA

This article is a well-written and thorough study. It describes six Omani families with children affected by ARPKD, highlighting the high consanguinity in this region, which makes a few PKHD1 mutations relatively common. The detailed analysis of potential structural disruptions in the PKHD1 protein is particularly impressive and adds significant value to the understanding of pathogenic mechanisms. Overall, the paper is clear, scientifically sound, and provides important insights. I strongly recommend this article for acceptance.

Is the work clearly and accurately presented and does it cite the current literature?

Yes

If applicable, is the statistical analysis and its interpretation appropriate?

Not applicable

Are all the source data underlying the results available to ensure full reproducibility?

Yes

Is the study design appropriate and is the work technically sound?

Yes

Are the conclusions drawn adequately supported by the results?

Yes

Are sufficient details of methods and analysis provided to allow replication by others?

Yes

Reviewer Expertise:

Genetics in polycystic kidney disease.

I confirm that I have read this submission and believe that I have an appropriate level of expertise to confirm that it is of an acceptable scientific standard.
